# Determinants of postnatal care non-utilization among women in Demba Gofa rural district, southern Ethiopia: a community-based unmatched case-control study

**DOI:** 10.1186/s12884-020-03244-9

**Published:** 2020-09-18

**Authors:** Markos Manote, Tsegaye Gebremedhin

**Affiliations:** 1 Sawla General Hospital, Gofa zone, Southern Nation Nationalities and Peoples’ Region, Ethiopia; 2 Department of Health Systems and Policy, Institute of Public Health, College of Medicine and Health Sciences, University of Gondar, P. O. Box: 196, Gondar, Ethiopia

**Keywords:** Determinants, postpartum care, non-utilization, Demba Gofa

## Abstract

**Background:**

Maternal and neonatal mortality remain a significant problem across much of the developing world, especially in sub-Saharan Africa countries. In Ethiopia, most maternal and neonatal deaths occur during the postpartum period; this is a critical time for monitoring the health of women and newborns, but the most neglected period for care. In rural communities of Ethiopia, the utilization of postnatal care service is very low and evidence on which factors contribute to the non-utilization of postnatal care (PNC) is insufficient. Consequently, this study was designed to identify the determinants of postnatal service non-utilization among women who gave birth in Demba Gofa rural district, Southern Ethiopia.

**Methods:**

A community-based unmatched case-control study was conducted among 186 cases (postnatal care non-utilizers) and 186 controls (postnatal care utilizers) in Demba Gofa rural district from March 1 to April 10, 2019. A previously tested interviewer-administered structured questionnaire was used for data collection. Binary logistic regression analysis was performed. In the final multivariable logistic regression analysis model, a p-value of less than 0.05 and an Adjusted Odd Ratio (AOR) with a 95% confidence interval (CI) was used to determine variables for postnatal care non-utilization.

**Results:**

In this study, women who delivered recently were incorporated within 186 cases and 186 controls. Not knowing the availability of PNC services (AOR: 4.33, 95% CI: 1.71–10.99), having a home delivery (AOR: 7.06, 95% CI: 3.71–13.44), ANC non-attendance (AOR: 6.14, 95% CI: 3.01–12.50), unable to make an independent decision (AOR: 9.31, 95% CI: 3.29–26.35), and not participating in the Women’s Development Army (WDA) (AOR: 5.09, 95% CI: 2.73–9.53) comprised the determinants which were assessed for non-utilization of postnatal care services.

**Conclusions:**

Encouraging institutional delivery along with integrated health education about postnatal care and postnatal danger signs, empowering women to execute independent decisions, accessing PNC services and strengthening participation in the Model Families will likely improve postnatal care service utilization in the district of Ethiopia.

## Background

Postnatal period, from birth to the first six weeks, is the most crucial period for survival in which the majority of maternal and newborn deaths are the highest, particularly in the first week [1, 2]. According to the World Health Organization (WHO), later to an uncomplicated vaginal birth in a health facility, mothers should receive care in the facility for at least 24 hours after birth [3] and for home delivery, the first postnatal contact should be as early as possible within 24 hours. Besides, an additional three postnatal contacts (on 48 to 72 hours, between 7th and 14th days, and six weeks after birth) are recommended for all mothers and newborns.

Globally, the maternal mortality ratio (MMR) was 216 maternal deaths per 100,000 live births in 2015, which occurred during pregnancy and childbirth [4]. The majority (99%) of the maternal deaths are from preventable causes occurring in low- and middle- income countries, including Ethiopia [5]. In many African countries, half of the maternal deaths occur during the first week after birth, and the majority of these happen during the first 24 hours because women and their newborns do not have access to care during the early postnatal period [1]. Ethiopia was one of the highest contributors in Africa with 412 per 100,000 live births of maternal mortality ratio in 2016. Besides, infant and neonatal mortalities were 48 and 29 per 1,000 live births, respectively, in which 61 and 58% of the materiality takes place in the postnatal period, correspondingly [6].

The health of mothers has been regarded as an indicator of the health of society [7]. The provision of services to the mother and their newborn has been set up for the prevention and reduction of maternal and neonatal deaths worldwide [5]. Postnatal care services help to safeguard women from complications and provide essential opportunities to assess the infant’s development [8]. Despite its importance, the postnatal period is generally the most neglected and underutilized services in developing countries, particularly Ethiopia, and most mothers do not receive care from a skilled health care provider during the first few days after delivery [3] [9]. A large proportion of maternal deaths occur during the first 48 hours after delivery that can be averted with crucial PNC services [7]. In developing countries, a fully functioning mother-baby package intervention has been estimated to have the postnatal cumulative effect of averting 75 to 85% of maternal death [10]. Lack of PNC utilization may result in a number of complications. Of those, maternal and neonatal infections, incomplete immunization, frequent and mistimed pregnancies, severe postpartum hemorrhage which claims at least 22% of maternal deaths and which kills a woman within two hours in the absence of medical interventions were identified [11].

According to the 2016 Ethiopian Demographic and Health Survey (EDHS) report, the national and Southern Nation Nationalities and Peoples’ Region (SNNPR) coverage of postnatal care service utilization within the first two days after delivery was only 17% and only 28% of deliveries were assisted by skilled attendants [10]. Considerably fewer mothers and newborns living in rural areas received postnatal care, compared to those living in urban areas (3% versus 32%) [12]. In Ethiopia, poor maternal and neonatal health outcomes are caused by a combination of factors, including the level and quality of health care services provided and the health-seeking behavior of mothers [13].

According to a few studies, literacy, ANC attendance, place of delivery of last-child, awareness about maternal complication and delivery complications that occurred during birth were factors associated with postnatal care service utilization [9, 13–16]. However, because of their cross-sectional study design and sociocultural setup, differences providing a general conclusion regarding the predictors of postnatal care utilization among mothers is limited. In particular, some of these studies were facility-based, utilized convenience sampling approach had a small sample size, recruited the participants through the under-five clinic, which only targeted mothers attending under-five clinics. Because of these, factors that motivate or hinder mothers from utilizing postnatal care services are not well established. Additionally, despite several studies on the utilization of postnatal care services, there are only a few studies to identify the factors that influence their non-utilization in Ethiopia. The implication is that women who need targeted interventions have been missed, and this would delay the country’s sustainable development goal (SDG) achievement. Therefore, this study designed to identify the determinants of postnatal care non-utilization in Demba Gofa district, southern Ethiopia.

## Methods

### Study design and settings

A community-based unmatched case control study was conducted in Demba Gofa rural district, Gofa zone, Southern Nation Nationalities and Peoples’ Region (SNNPR), Ethiopia, from March 1 to April 10, 2019. Demba Gofa district is located 515 km to the southwest of Addis Ababa, the capital of Ethiopia and 284 km from Hawassa, the capital of SNNPR. Administratively, the district is divided into 34 kebeles (the smallest administrative unit) with a total population of 96,433 (projected from 2007 census) in the year 2018. Of these, 49,180 were female; 22,468 of the women were in the reproductive age group and 1,630 were gave birth in the last six months. Moreover, the district has four health centers, 34 health posts, and 13 private clinics.

All women who gave birth in 2018 in the district were the source population of the study, whereas women who delivered from July 1 to December 31, 2018 in the selected kebeles of the district were the study population. Mothers who were too sick to participate were excluded from the study.

### Sample size and sampling procedures 

The sample size was calculated using a power approach through Epi-Info version 7 software and assuming a 33.5% proportion of postnatal services utilization [17], 80% power, 95% confidence level (CI), one-to-one ratio, 1.5 design effect, and 10% non-response rate that yielded a sample of 376 (188 cases and 188 controls).

Initially, 14 kebeles (40% of the total kebeles) were selected using the lottery method [18]. The survey was conducted to obtain PNC service utilizers and non-utilizers in the last six months (July 1 to December 31, 2018). A sampling frame was prepared for cases and controls separately from the health post-registration. Then, the sample was proportionally allocated to the 14 kebeles based on the estimated number of mothers, who gave birth for the last six months. The final participants were selected using the simple random sampling technique (lottery method) from the delivery registries of the health posts. Then, home visits and interviews were conducted using household numbers.

### Variables and measurements

Postnatal care non-utilization was the dependent variable of this study. Cases were women who did not use PNC services from health care providers within 42 days of childbirth. For uncomplicated vaginal delivery in the health facility, a mother discharged before 24 hours is considered as non-utilizer unless she gets an additional visit within 42 days of delivery [19, 20]. Controls were women who utilized postnatal services, which was measured if the mothers received one of the postnatal services; immunization, family planning and health education on childcare, breastfeeding, physiotherapy, physical examination, treatment, and counseling service [10, 21].

The independent variables of the study were sociodemographic and economic factors, sociocultural factors, knowledge and attitude related factors, health system-related factors, and reproductive and obstetric related factors were the independent variables of the study.

Sociodemographic and economic factors were age, family size, educational status of the mother and her husband, marital status, religion, occupational status of the mother and her husband, and household wealth status. The wealth status of the household was measured using 21 household assets taken from the EDHS 2016 and analysis using principal component analysis [6, 22].

Sociocultural factors were cultural seclusion and decision making. Cultural seclusion is a cultural/social practice that forbids mothers from going out or joining peoples for 40 days, starting from the day of delivery [23]. Decision making is the ability of the mother to make and execute independent decision about PNC service utilization.

Knowledge and attitude on postnatal services were knowledge on the availability of postnatal health services, knowledge on delivery complications, attitude towards postnatal services, attitude to health service providers. Postpartum danger sign awareness was measured when a mother mentioned at least one postpartum complication of mother and newborn occur after birth [24, 25]. Attitude about PNC service was the total score of a way of thinking about PNC service or behaving towards the use of the service to the health of the mother, her child, and recommending the service to others. The score on the Likert scale less than the mean was considered as favorable and scored higher than the mean as an unfavorable attitude at the individual level [24].

Health system-related factors were distance to the nearest health facility, participation in HDA, model family graduation, perceived quality of postnatal services, cost of the service and transportation, and waiting time. Model family; a family having women who are graduated after attending 15 days of training and implemented health extension packages [7, 26]. Distance from the nearest health facility was measured using the time taken to reach the facility on foot [24]. Perceived quality of care was the composition of women’s perception, which was measured with a five-point Likert scale of 10 services quality measurement items. Finally, the score greater than the mean were considered as poor and less than the mean as good quality [27, 28].

Reproductive and obstetric related factors were ANC follow up, parity, place of delivery, mode of delivery, desire for pregnancy, birth size, and history of abortion. A desire for pregnancy refers to whether the mother wanted, wanted later, or wanted not more the child at conception [22].

### Data collection tools and procedures 

An interviewer-administered standardized structured questionnaire was developed and used after reviewing other similar studies [6, 10, 29–34]. The questionnaire has consisted of a range of variables; sociodemographic and cultural, socio-economic, and knowledge, attitudes, and health system-related characteristics. The tool was initially developed in English and translated into the local language (Goofatho) and finally back to English to ensure consistency. Eight trained diploma nurses and two public health officers were recruited from Sawla general hospital (the nearby hospital) for data collectors and supervisors, respectively. A pretest was conducted on 5% of the sample size in Geze Gofa (one of the neighboring districts with similar characteristics) and the necessary amendment was done before the actual data collection. The principal investigator and supervisors checked data accuracy, consistency and completeness daily.

### Data management and analysis 

The completed data were entered into Epi-Data version 3.1 software and exported to Statistical Package for Social Science (SPSS) version 23 for analysis. Both descriptive and binary logistic regression analysis was done. Variables with p-values of less than 0.2 in the bivariable logistic regression were candidates for the multivariable logistic regression analysis after checking model fitness by Hosmer-Lemeshow goodness of fit test, chi-square, and multi-collinearity assumptions. In the final multivariable logistic regression analysis model, a p-value of less than 0.05 and adjusted odds ratio (AOR) with a 95% confidence interval (CI) were used to identify the determinants of postnatal care non-utilization.

## Results

### Sociodemographic and economic and cultural characteristics of participants 

A total of 372 (186 cases and 186 controls) mothers were responded to the interviewer-administered questionnaire with an overall response rate of 98.9%. The mean age of mothers among cases and controls was 25.6 (SD ± 6) and 26.0 years (SD ± 5.97), respectively. Ninety-three and ninety-five percent of cases and controls were married, respectively. Educationally, 31.2% of cases and 17.2% of controls were unable to read and write. Regarding occupation, 90.9% of cases and 79.0% controls were housewives. Thirty-one percent of cases’ and seventeen percent of controls’ husbands were unable to read and write. Nearly half of the cases and thirty-one percent of controls were in the poor wealth status. Almost sixteen percent of cases and controls reported that their culture prohibited them from attending PNC services. Regarding decision making, 50% of cases and 72% of controls were decided jointly with their spouse about PNC service utilization (Table 1).

**Table 1:** Sociodemographic and economic characteristics of participants in Demba Gofa rural district, Southern Ethiopia, May 2019

Variables	Categories	Cases (*n* = 186)	Controls (*n* = 186)
		n (%)	n (%)
Age in years	15–19	32 (17.2)	30 (16.1)
	20–24	45 (24.2)	47 (25.3)
	25–29	55 (29.6)	53 (28.5)
	30–34	35 (18.8)	34 (18.3)
	>=35	19 (10.2)	22 (11.9)
Marital status	Married	173 (93.0)	178 (95.7)
	Divorced	7 (3.8)	3 (1.6)
	Single	6 (3.2)	5 (2.7)
Ethnicity	Gofa	182 (97.8)	171 (91.9)
	Gamo	1 (0.5)	9 (4.8)
	Others^a^	3 (1.6)	6 (3.3)
Religion	Protestant	160 (86.0)	162 (87.1)
	Orthodox	25 (13.4)	21 (11.3)
	Muslim	1 (0.5)	3 (1.6)
Maternal educational status	Unable to read and write	66 (35.5)	39 (21.0)
	Primary	87 (46.8)	95 (51.1)
	Secondary and above	33 (17.7)	52 (28.0)
Husband educational status	Unable to read and write	58 (31.2)	32 (17.2)
	Primary	83 (44.7)	85 (45.7)
	Secondary and above	45 (24.2)	69 (37.1)
Mother’s occupation	Housewife	169 (90.9)	147 (79.0)
	Merchant	15 (8.1)	25 (13.4)
	Others^b^	2 (1.0)	11 (7.5)
Husband’s occupation	Farmer	142 (76.3)	118 (63.4)
	Merchant	17 (9.1)	18 (9.7)
	Government employee	8 (4.3)	21 (11.3)
	Others^c^	9 (10.2)	29 (15.6)
Wealth status	Richest	27 (14.5)	48 (25.8)
	Rich	29 (15.6)	45 (24.2)
	Middle	38 (20.4)	36 (19.4)
	Poor	42 (22.6)	29 (15.6)
	Poorest	50 (26.9)	28 (15.1)
Decision making about PNC services	Jointly	93 (50.0)	134 (72.0)
	Self	29 (15.6)	36 (19.4)
	Husband	35 (18.8)	10 (5.4)
	Others^d^	29 (15.6)	6 (3.2)

^a^Wolaita, Amhara; ^b^Government employee, maidservant, daily labour, and student; ^c^Daily labour and student; ^d^Husband, mothers, mother in law, friends, and neighbours

### Knowledge, attitude and health system-related characteristics 

Of the total participants, 45.2% of cases and 9.1% of controls did not hear about PNC. Moreover, 66.1% of cases and 26.9% of controls did not know any danger sign of the postnatal period. Regarding service availability, 34.4% of cases and 4.8% of controls do not know the availability of the service in their nearest health facility. Three-fourths of cases and 94.1% of controls had a favorable attitude towards PNC services utilization. Regarding distance to the nearest health facility, 154 (82.8%) of cases and 172 (92.5%) of controls travel less than an hour on foot to reach the nearest health facility. Eighty-four and fifty-two percent of the cases and controls were not graduated from the model family training given by health extension workers in the community (Table 2).

**Table 2:** Knowledge, attitude, and health system-related factors of participants in Demba Gofa rural District, Southern Ethiopia, May 2019

Variables	Categories	Cases (*n* = 186)	Controls (*n* = 186)
		n (%)	n (%)
Know the complications during delivery	No	111 (59.7)	78 (41.9)
	Yes	75 (40.3)	108 (58.1)
Know the availability of PNC service	No	64 (34.4)	9 (4.8)
	Yes	122 (65.6)	177 (95.2)
Attitude towards PNC services	Unfavorable	26 (14.0)	3 (1.6)
	Medium	20 (10.8)	8 (4.3)
	Favorable	140 (75.3)	175 (94.1)
Distance to nearest health facility	< 1 hour	154 (82.8)	172 (92.5)
	>=1hours	32 (17.2)	14 (7.5)
Participation in HDA	Yes	69 (37.1)	151 (81.0)
	No	117 (62.9)	35 (19.0)
Graduated in model family training	Yes	29 (15.6)	89 (47.8)
	No	157 (84.4)	97 (52.2)

HDA: Women health development army

### Reproductive and obstetric characteristics 

Forty-one percent of cases and 43.5% of controls were multiparas. Of the total participants, 12.9% of cases and 17.2% of controls had a history of abortion. Regarding ANC visits, 65.1 and 14.0% of cases and controls did not received ANC during their last pregnancy, respectively. Sixty-eight percent of cases and 23.7% of controls gave their recent birth at home. Regarding the mode of delivery, 95.7% of cases and 84.9% of controls have delivered spontaneously vaginally. Furthermore, 57.5% of cases and 69.9% of controls had a plan to get pregnancy of their last child then (Table 3).

**Table 3:** Reproductive and obstetric characteristics of participants in Demba Gofa rural district, Southern Ethiopia, May 2019

Variables	Categories	Cases (*n* = 186)	Controls (*n* = 186)
		n (%)	n (%)
ANC follow-up	No visit	121 (65.1)	26 (14.0)
	Once	16 (8.6)	13 (7.0)
	Twice	19 (10.2)	34 (18.4)
	Three times	16 (8.8)	53 (28.4)
	>=4 times	14 (7.3)	60 (32.2)
Abortion history	No	162 (87.1)	154 (82.8)
	Yes	24 (12.9)	32 (17.2)
Place of delivery	Home	126 (67.7)	44 (23.7)
	Health institution	60 (32.3)	142 (76.3)
Mode of delivery	Spontaneous delivery	178 (95.7)	158 (84.9)
	Instrumental delivery	8 (4.3)	28 (15.1)
Desire to pregnancy	Wanted no more	40 (21.5)	37 (19.9)
	Wanted later	39 (21)	19 (10.2)
	Wanted then	107 (57.5)	130 (69.9)

### Determinants of postnatal care non-utilization

In the multivariable logistic regression analysis, women being unable to make an independent decision, women not know the availability of PNC services, home delivery, not participating in HDA, and no ANC follow up were the determinants of postnatal care services non-utilization.

Accordingly, mothers whose postnatal services utilization decided by their husbands were 9.31 times more likely not to utilize the postnatal services compared to those who decided by themselves (AOR: 9.31, 95% CI: 3.29–26.35). The odds of PNC care non-utilization was four times more likely among women who not know the availability of PNC services compared to their counterparts (AOR: 4.34, 95% CI: 1.71–10.99). Mothers who did not attend ANC were 6.14 times more likely not to utilize postnatal care compared to those who attend ANC (AOR: 6.14, 95% CI: 3.01–12.51). The odds of PNC care non-utilization was seven times higher among women who gave birth at home compared to those who gave birth at the health institution (AOR: 7.106, 95% CI: 3.71–13.44). The odds of PNC care non-utilization was five times higher among women who were not participating in the HDA network compared to women who participate (AOR: 5.10, 95% CI: 2.73–9.53) (Table 4).

**Table 4:** Bivariable and multivariable logistic regression analysis for determinants of postnatal care service non-utilization among mothers in Demba Gofa rural District, Sothern Ethiopia, May 2019

Variables	Cases (*n *= 186)	Controls (*n* = 186)	COR (95%CI)	AOR (95% CI)
	n (%)	n (%)		
Educational level of mother				
Unable to read & write	66 (352.5)	39 (21)	2.67(1.48–4.81)	0.48 (0.17–1.31)
Primary	87 (46.8)	95 (51.1)	1.46 (0.85–2.44)	0.53 (0.23–1.21)
Secondary and above	33 (17.7)	52 (28.0)	1	1
Educational level of husband				
Unable to read & write	58(31.2)	32 (17.2)	2.78 (1.57–4.92)	0.50 (0.19–1.34)
Primary education	83(44.7)	85 (45.7)	1.50 (0.92–2.42)	0.52 (0.23–1.18)
Secondary and above	45 (24.2)	69 (37.1)	1	1
Occupation of husband				
Farmer	142(76.3)	118 (63.4)	1	1
Merchant	17(9.1)	18 (9.7)	0.78 (0.14–1.15)	0.29 (0.06–1.37)
Government employee	8 (4.3)	21 (11.3)	0.32 (0.63–2.58)	0.57 (0.19–1.69)
Housewife	19(10.2)	29 (15.6)	0.54 (0.29–1.67)	0.39 (0.11–1.44)
Decision making				
Self	29 (15.6)	36(19.4)	1	1
Jointly	93(50)	134(72.0)	0.86 (0.49–1.50)	1.41 (0.62–3.20)
Husband	64(30.1)	16(8.6)	4.96 (2.31–0.65)	9.31 (3.29–6.35) *
Wealth status				
Poorest	50(26.9)	28 (15.1)	3.17 (1.64–6.15)	0.59 (0.23–1.52)
Poor	42(22.6)	29 (15.6)	2.57 (1.32–5.02)	1.28 (0.49–3.37)
Middle	38(20.4)	36 (19.4)	1.88 (0.97–3.62)	1.52 (0.57–4.17)
Rich	29(15.6)	45 (24.2)	1.15 (0.59–2.22)	1.31 (0.51–3.38)
Richest	27(14.5)	48 (25.8)	1	1
Know delivery complication				
No	111(59.7)	78 (41.9)	2.05(1.36–3.10)	0.57 (0.29–1.11)
Yes	75(40.3)	108 (58.1)	1	1
Know PNC service availability				
No	64(34.4)	9 (4.8)	10.32 (4.95 − 0.51)	4.34 (1.71–0.99) *
Yes	122(65.6)	177 (95.2)	1	1
Attitude				
Unfavorable	26 (14)	3 (1.6)	10.83 (3.21-0.53)	0.77 (0.15–3.97)
Medium	20 (10.8)	8 (4.3)	3.12 (1.34–7.31)	0.74 (0.10–5.25)
Favorable	140(75.3)	175 (94.1)	1	1
Perception of provider				
Selfish	12 (6.5)	7 (3.8)	2.07(0.79–5.36)	3.57 (0.69–18.40)
Rude	29 (15.6)	5 (2.7)	6.96 (2.63–18.44)	0.84 (0.16–4.31)
Friendly	145 (78)	174 (93.5)	1	1
ANC follow up				
No	121 (65.1)	26 (14.0)	11.46 (6.86 − 0.12)	6.14 (3.01–2.51) *
Yes	65 (34.9)	160 (86.0)	1	1
Desire for pregnancy				
Wanted no more	40 (21.5)	37 (19.9)	1.31(0.78–2.20)	1.16 (0.45–2.98)
Wanted later	39(21)	19 (10.2)	2.49 (1.36–4.57)	0.56 (0.22–1.41)
Wanted then	107(57.5)	130 (69.9)	1	1
Mode of delivery				
Spontaneous	178 (95.7)	158 (84.9)	3.94 (1.75–8.90)	3.29 (0.78–13.89)
Instrumental	8 (4.3)	28 (15.1)	1	1
Abortion history				
No	162(87.1)	154 (82.8)	1.40 (0.79–2.49)	1.75 (0.70–4.36)
Yes	24(12.9)	32 (17.2)	1	1
Place of delivery				
Home	126(67.7)	44(23.7)	6.78(4.29–0.70)	7.11 (3.71–3.44) *
Health institution	60 (32.3)	142(76.3)	1	1
Distance to the nearest health facility				
< 1 hour	154(82.8)	172 (92.5)	1	1
>=1hours	32(17.2)	14 (7.5)	2.55 (1.31–4.96)	0.55 (0.16–1.89)
Cost of service and transportation				
Big problem	10 (5.4)	4 (2)	2.92(1.26–7.19)	1.07 (0.43–2.62)
Medium	52 (28)	37 (20)	1.64(0.96–2.52)	2.92 (0.34–25.46)
No problem	124(66.7)	145(78)	1	1
Waiting time				
< 1 hour	141 (75.8)	161 (86.6)	1	1
1–2 hours	29 (15.6)	20 (10.8)	1.65(0.91–3.10)	0.86 (0.26–2.84)
> 2 hours	16 (8.6)	5 (2.7)	3.65 (1.31 − 0.23)	1.90 (0.21–17.7)
Quality of service				
Poor	20 (10.8)	5 (2.7)	5.32 (1.95–4.56)	0.90 (0.26–3.16)
Medium	36 (19.4)	8 (4.3)	5.98(2.69–3.31)	1.75 (0.26–11.70)
Good	130 (69.9)	173 (93)	1	1
Participation in HDA				
No	69 (37.1)	151 (81)	7.32 (4.56–1.74)	5.10 (2.73–9.53) *
Yes	117 (62.9)	35 (19)	1	1

*AOR *Adjusted Odds Ratio, *CI *Confidence Interval, *COR *Crude Odds Ratio, 1: Reference category, *statistically significant at* p*-value < 0.05

## Discussion

Our findings showed that decisions for postnatal service utilization made by their husbands, not knowing the availability of PNC services, not attended ANC, home delivery, and HAD no participation were the determinants of postnatal care non-utilization.

The finding showed that the odds of PNC care non-utilization among those women’s decisions made by their husbands were nine times more compared to those decided by themselves. The finding is supported by studies conducted in Gondar zuria district [11] and Jabitena district, Amhara region [35]. The finding is also in agreement with a study conducted in that of Mangochi district, Malawi [13]. The possible reason might be those women who are unable to make independent decisions and have a dependency on husbands about their health could miss the opportunity to contact the health professionals, develop a low level of awareness about PNC services. However, the finding is in contradict with a study in the pastoral communities of Afar, Ethiopia [36]. This disagreement can be justified by the variations in the study area with critical cultural beliefs, attitudes and practice in that of Afar region. So, those women made a decision by themselves might go to the traditional healers rather than seeking to the health factifies. Besides, husbands are mode dominates and the presence of low women empowerment can justify the disagreement.

In this study, the odds of PNC care non-utilization was about four times more likely among women who not know the availability of PNC services compared to those who know the availability of PNC services. This finding is also supported by a study conducted in Mangochi district, Malawi [13]. The possible justification can be women who not know the availability of PNC services would not be motivated to use the service and might not seek to get services in the postpartum period.

Our study finding showed that women who did not attend ANC had six times more chance of not getting postnatal services utilization compared to mothers who were attended ANC. This result was comparable with those of studies finding in Diga, Amigna, and Lemo district, Ethiopia [16, 24, 34]. Moreover, the findings were similar to that of a study fining in Uganda [37] and Mangochi district, Malawi [13]. This finding could be explained by the fact that women who did not have ANC follow up might not get information about the benefits of postnatal services for herself and her baby.

The study finding showed that the odds of PNC care non-utilization was seven times higher among women who gave birth at home compared to those who gave birth in the health facility. The result is in agreement with studies conducted in Gondar zuria, Amhara region [11], and Diga district, Oromiya region [24]. This finding is also supported by a study conducted in Nigeria [22]. The possible reason could be if a mother gave birth at home, the adequacy of receiving advice and knowing the postnatal care benefits is low that can significantly reduce the postnatal services seeking behavior. This might be attributed to the fact that women who gave their last birth at home have lesser opportunities to get exposed to health education-related to PNC services at the time of delivery and thus get no access to learn about the types, benefits, and availabilities of PNC services.

Our finding showed that the odds of PNC care non-utilization was five times higher among women who were not participating in the HDA network compared to their counterparts. Even though there is no study indicating the association between postnatal care service utilization and model family graduation, it is in agreement with a study conducted in Abuna Gindeberet, Oromiya region [38] that showed model family significantly increased maternal services utilization. The possible explanation could be women who do not participate in HDA networks might have lesser exposure to the theoretical and practical knowledge related to maternal health services; hence, they would be less likely to be motivated to use postnatal care services. Furthermore, women might miss health education, which has been provided by the health extension workers at the community level if they were not participating in the HDA networks.

### Strength and limitation of the study 

Our study might address the real determinants of postnatal services non-utilization since it is a community-based case-control study. The source of data for this study was based on the self-reporting of respondents; this might have some limited validation of information obtained from the subjective source. Moreover, the women might have experienced recall bias, particularly regarding the services they received during their previous postnatal services, for instance. Compared to other studies, however, our work assessed later events that preceded the study by only six months.

## Conclusions

This study revealed that home delivery, not know the availability of PNC services, ANC non-attendance, unable to make independent decisions about postnatal service utilization, and not participating in HDA networks were found to be the determinants of PNC service non-utilization. Therefore, encouraging ANC, institutional delivery, along with integrated and improving awareness on postnatal care services, empowering women to execute independent decisions about their PNC utilization, and strengthening participation in HDA will improve postnatal care service utilization in the districts of Ethiopia.

## Abbreviations

ANC: Ante Natal Care
AOR: Adjusted Odds Ratio
CI: Confidence Interval
EDHS: Ethiopian Demographic Health Survey
HAD: Health Development Army
LMIC: Low-and-Middle Income countries
PNC: Postnatal Care
SNNPR: South Nation Nationality and Peoples Region
SPSS: Statistical Packages for Social Sciences
WHO: World Health Organization

## Availability of data and materials

Data will be available upon reasonable request from the corresponding author.

## References

[1] Warren C, Daly P, Toure L, Mongi P. Opportunities for Africa’s newborns: Postnatal care. 2006. In.; 2015.

[2] World Health Organization. Postnatal care for mothers and newborns highlights from the WHO 2013 guidelines. Geneva: WHO; 2015.

[3] World Health Organization. WHO recommendations on postnatal care of the mother and newborn. Geneva: World Health Organization; 2014. 24624481

[4] World Health Orgnization. Trends in maternal mortality: 1990-2015: estimates from the WHO, UNICEF, UNFPA, World Bank group and the UnitedNations Population Division. Geneva: World Health Organization; 2015.

[5] World Health Orgnization. World health statistics 2016: monitoring health for the SDGs. Geneva: World Health Organization; 2016.

[6] Central Statistical Agency (CSA) [Ethiopia] and ICF. Ethiopia Demographic and Health Survey 2016. Addis Ababa, Ethiopia, and Rockville, Maryland,USA: CSA and ICF; 2016. https://dhsprogram.com.

[7] Federal Ministry of Health E. Health sector transformation plan 2015/16-2019/2020. In.; 2015.

[8] Mwangi A, Warren CA, Koskei N, Blanchard H. "Strengthening postnatal care services including postpartum family planning in Kenya," FRONTIERS Final Report. Washington, DC: Population Council; 2008.

[9] Aseweh Abor P, Abekah-Nkrumah G, Sakyi K, Adjasi CK, Abor J (2011). The socio-economic determinants of maternal health care utilization in Ghana. Int J Soc Econ.

[10] Amane T, Deme A, Elile F, Megersa N, Tsegaw Y, et al. Assessment of Postnatal Care Service Utilization and Associated Factors in Asella Town, Arsi Zone, Oromiya Regional State, Ethiopia. Glob J Reprod Med. 2018;6(1):555678. 10.19080/GJORM.2018.06.555678.

[11] Tesfahun F, Worku W, Mazengiya F, Kifle M (2014). Knowledge, perception and utilization of postnatal care of mothers in Gondar Zuria District, Ethiopia: a cross-sectional study. Matern Child Health J.

[12] Keyes EB, Haile-Mariam A, Belayneh NT, Gobezie WA, Pearson L, Abdullah M, Kebede H (2011). Ethiopia’s assessment of emergency obstetric and newborn care: Setting the gold standard for national facility‐based assessments. International Journal of Gynecology Obstetrics.

[13] Sagawa J. Determinants of postnatal care utilization among mothers in Mangochi District, Malawi. 2016. http://www.dspace.mak.ac.ug/handle/10570/5636. 10.1186/s12884-021-04061-4 PMC8406845 34461844

[14] Yarinbab TE, Tona WC: Utilization of Postnatal Care and its Determinants in Loma District, Southwest Ethiopia: a Community Based Cross Sectional Study. 2017.

[15] Limenih MA, Endale ZM, Dachew BA. Postnatal Care Service Utilization and Associated Factors among Women Who Gave Birth in the Last 12Months prior to the Study in Debre Markos Town, Northwestern Ethiopia: A Community-Based Cross-Sectional Study. Int J Rep Med. 2016;2016:7095352. 10.1155/2016/7095352 PMC4940555 27433481

[16] Belachew T, Taye A, Belachew T: Postnatal care service utilization and associated factors among mothers in Lemo Woreda, Ethiopia. J Women’s Health Care 2016, **5**(10.4172):2167-0420.1000318.

[17] Limenih MA, Endale ZM, Dachew BA (2016). Postnatal Care Service Utilization and Associated Factors among Women Who Gave Birth in the Last 12 Months prior to the Study in Debre Markos Town, Northwestern Ethiopia: A Community-Based Cross-Sectional Study. International Journal of Reproductive Medicine.

[18] Sambo L, Chatora R, Goosen E. Tools for assessing the operationality of district health systems. Brazzaville: World Health Organization, RegionalOffice for Africa, Brazzaville; 2003.

[19] Organization WH: Postnatal care for mothers and newborns: Highlights from the World Health Organization 2013 Guidelines. Avaible from: http://www.whoint/maternal_child_adolescent/publications/WHOMCA-PNC*-2014-Briefer_ A* 2015, 4.

[20] Titaley CR, Dibley MJ, Roberts CL (2009). Factors associated with non-utilisation of postnatal care services in Indonesia. Journal of Epidemiology Community Health.

[21] Tesfaye S, Barry D, Gobezayehu AG, Frew AH, Stover KE, Tessema H, Alamineh L, Sibley LM (2014). Improving coverage of postnatal care in rural Ethiopia using a community-based, collaborative quality improvement approach. J Midwifery Women Health.

[22] Somefun OD, Ibisomi L (2016). Determinants of postnatal care non-utilization among women in Nigeria. BMC Res Notes.

[23] Akibu M, Tsegaye W, Megersa T, Nurgi S. Prevalence and Determinants of Complete Postnatal Care Service Utilization in Northern Shoa, Ethiopia. J Preg. 2018;2018:8625437. 10.1155/2018/8625437 PMC6112074 30186633

[24] Heyi WD, Deshi MM, Erana MG. Determinants of postnatal care service utilization in diga district, east wollega zone, wester Ethiopia: case-control study. EJRH. 2018;10(4):52–61.

[25] Hailu M, Gebremariam A, Alemseged F. Knowledge about Obstetric Danger Signs among Pregnant Women in Aleta Wondo District, Sidama Zone, Southern Ethiopia. Ethiopian J Health Sci. 2010;20(1):25. 10.4314/ejhs.v20i1.69428 PMC3275898 22434957

[26] Gebru T, Taha M, Kassahun W (2014). Risk factors of diarrhoeal disease in under-five children among health extension model and non-model families in Sheko district rural community, Southwest Ethiopia: comparative cross-sectional study. BMC Public Health.

[27] Kanté AM, Chung CE, Larsen AM, Exavery A, Tani K, Phillips JF (2015). Factors associated with compliance with the recommended frequency of postnatal care services in three rural districts of Tanzania. BMC Pregnancy Childbirth.

[28] Emelumadu OF, Onyeonoro UU, Ukegbu AU, Ezeama NN, Ifeadike CO, Okezie OK (2014). Perception of quality of maternal healthcare services among women utilising antenatal services in selected primary health facilities in Anambra State, Southeast Nigeria. Nigerian medical journal: journal of the Nigeria Medical Association.

[29] Angore BN, Tufa EG, Bisetegen FS (2018). Determinants of postnatal care utilization in urban community among women in Debre Birhan Town, Northern Shewa, Ethiopia. Journal of Health Population Nutrition.

[30] Olajubu AO, Olowokere AE, Ogundipe MJ, Olajubu TO. Predictors of Postnatal Care Services Utilization Among Women in Nigeria: A Facility‐Based Study. J Nurs Sch. 2019;51(4):408-16. 10.1111/jnu.12473 30919580

[31] Kebede SD, Roba KT, Assefa N, Munye T, Alebachew W (2019). Prevalence and Predictors of Postpartum Care Uptake Among Mothers Who Gave Birth in the Last Six Months in Mertule Mariam District Northwest Ethiopia. Am J Nurs.

[32] Wudineh KG, Nigusie AA, Gesese SS, Tesu AA, Beyene FY (2018). Postnatal care service utilization and associated factors among women who gave birth in Debretabour town, North West Ethiopia: a community-based cross-sectional study. BMC Pregnancy Childbirth.

[33] Shukure R, Mohammed H, Wudmetas A, Mohammed S (2018). Assessment of Knowledge and Factors Affecting Utilization of Postnatal Care in Fiche Town, Oromia Region, Ethiopia. International Journal of Clinical Dermatology.

[34] Kifle A, Sena L, Jarso H (2018). Determinants of Postnatal Care Service Utilization, Amigna District, Arsi Zone, Southeast Ethiopia: A Case-Control Study. J Women’s Health Care.

[35] Workineh YG, Hailu DA (2014). Factors affecting utilization of postnatal care service in Jabitena district, Amhara region, Ethiopia. Sci J Public Health.

[36] Yousuf J, Ayalew M, Seid F (2011). Maternal health beliefs, attitudes and practices among Ethiopian Afar. Exchange.

[37] Nankwanga A: Factors influencing utilisation of postnatal services in Mulago and Mengo hospitals Kampala, Uganda. University of the Western Cape; 2004.

[38] Gela BD, Bedada ND, Jaleta FT, Sinkie SO (2014). Antenatal care utilization and associated factors from rural health extension workers in Abuna Gindeberet district, West Shewa, Oromiya region, Ethiopia. American Journal of Health Research.

